# Microscopy

**Published:** 1888-09

**Authors:** 


### Microscopy.

The indiscriminate use of the terms "photo-micrograph" and "micro-photograph" has led to much confusion, not only among the non-scientific, but also among microscopists. It seems to have originated in England about the year 1850. In a recent number of a journal the following obscure definition is given: "A photo-micrograph is a macroscopic photograph of a microscopic object; a micro-photograph is a microscopic photograph of a microscopic object." In simple language, the meaning is this: A photo-micrograph is a large photograph of an original small object, whilst a micro-photograph is a small picture of a large object. The pictures which we frequently see in small watch charms and handles of pocket knives are micro-photographs.

PATENT MEDICINE MAN (to editor)—"You made a nice mess of that testimonial advertisement." *Editor*—"How?" *Patent Medicine Man*—"John Smith wrote: 'Your Live Forever Pellets are doing me a great deal of good. Send me another box;' and I told you to give it a prominent place." *Editor*—"I did—immediately preceding the death-notices." *Patent Medicine Man*—"Yes, and the first death-notice on the list was that of John Smith."—*Tit-Bits.*

HOW IT WORKS.—Bobley—I hear they've been trying the faith-cure on Jawkins. Wiggins—Yes, it's a great thing for rheumatism. Bobley—Indeed! Is he stronger? Wiggins—No; but the rheumatism is. It's got him all twisted up in a hard knot now.—*Judge.*

